# New Therapies for Advanced Thyroid Cancer

**DOI:** 10.3389/fendo.2020.00082

**Published:** 2020-05-22

**Authors:** Diprajan Laha, Naris Nilubol, Myriem Boufraqech

**Affiliations:** Surgical Oncology Program, National Cancer Institute, National Institutes of Health, Bethesda, MD, United States

**Keywords:** thyroid cancer, targeted therapies, advanced thyroid cancer, RAI-refractory, immunotherapy

## Abstract

Thyroid cancer is the most common endocrine cancer. The discovery of new biomarkers for thyroid cancer has significantly improved the understanding of the molecular pathogenesis of thyroid cancer, thus allowing more personalized treatments for patients with thyroid cancer. Most of the recently discovered targeted therapies inhibit the known oncogenic mechanisms in thyroid cancer initiation and progression such as MAPK pathway, PI3K/Akt-mTOR pathways, or VEGF. Despite the significant advances in molecular testing and the discoveries of new and promising therapeutics, effective treatments for advanced and metastatic, iodine-refractory thyroid cancer are still lacking. Here, we aim to summarize the current understanding of the genetic alterations and the dysregulated pathways in thyroid cancer and to discuss the most recent targeted therapies and immunotherapy for advanced thyroid cancer with a promising anti-tumor activity and clinical benefit.

## Introduction

Thyroid cancer is originating from follicular epithelial cells or parafollicular C cells. Follicular cells-derived thyroid cancers are classified into 4 histological types: papillary thyroid cancer (PTC 80–85%), follicular thyroid cancer (FTC 10–15%), poorly-differentiated thyroid cancer (PDTC, <2%), and anaplastic thyroid cancer (ATC, <2%). PTC and FTC are well-differentiated thyroid cancers (WDTC) accounting for most of thyroid cancer cases. The genetic alterations associated with differentiated thyroid cancer include, but are not limited to, *BRAF, RAS* mutations, or *RET/PTC* rearrangements which leads to the activation of MAPK oncogenic pathway. The existence of well-differentiated or poorly differentiated foci within an undifferentiated tumor suggests a progressive dedifferentiation process of WDTC that leads to ATC. *BRA*F^V600E^ mutation and *RAS* mutations are detected in 23% and 20% of ATC, respectively, however additional co-occurrence of *TP53* and *TERT* promoter mutations with known driver mutations are only common in ATC. These findings suggest that additional pathogenic alterations in tumor suppressors and oncogenes can lead to progression of WDTC to PDTC or ATC.

Surgical resection is the standard management for most patients with thyroid cancer. Patients with low-risk WDTC can be treated with surgery alone while those with high-risk features may require thyrotropin (TSH) suppression and radioiodine therapy (RAI) (1). Although anaplastic thyroid cancer (ATC) is the most aggressive histological subtype of thyroid cancer, most thyroid cancer-related deaths are due to the progression of radioactive-iodine refractory WDTCs. The poor survival of these patients with advanced thyroid cancer shows the lack of effective therapeutic strategies.

In this review, we summarized all the new therapeutic strategies that showed response in advanced thyroid cancer.

## A-Classification of Thyroid Cancer

### Papillary Thyroid Carcinoma

PTC is the most common type of thyroid carcinoma of follicular cell origin and accounts for 80% of all WDTC. PTC is driven by activating mutations in *BRAF, RAS*, or *RET/PTC* rearrangements which activates thyroid cell abnormal proliferation. Initial management of WDTC includes surgical resection, radioactive iodine ablation (RAI) and thyroid-stimulating hormone (TSH) suppression. Although thyroid cancer has a good prognosis, 10–15% of patients with thyroid cancer have recurrent disease, and 5% will develop distant metastasis (lungs and bones) and cancer specific-death occurs in some cases.

Analysis of morphological and pathological features of PTC in association with the clinical outcome of patients with PTC has identified several variants of papillary thyroid cancer: 1- classical variant of PTC (50–60% of PTC cases), is a low-risk subtype with an excellent prognosis, 2- Tall-cell variant of PTC (5–11% of all PTC cases) is more aggressive than cPTC with a higher risk of locoregional and distance metastasis, recurrence, and mortality rates (2, 3), 3- Follicular variant of PTC (FVPTC) (24-33% of PTC cases) are divided into invasive encapsulated FV-PTC and non-invasive encapsulated FV-PTC, also known as non-invasive follicular thyroid neoplasm with papillary-like nuclear features (NIFTP). NIFTP are classified as non-cancerous lesions with favorable clinical behavior (4). Rare histologic variants of PTC include solid, diffuse sclerosing, columnar, trabecular, oncocytic, and hobnail. These variants are associated with high rates of metastasis and thyroid cancer-related mortality (3, 5) and thus, are sometimes categorized as poorly differentiated thyroid cancer (PDTC).

### Follicular Thyroid Carcinoma

Follicular thyroid carcinoma (FTC) is the second most common thyroid cancer after papillary carcinoma. Follicular carcinoma is also well-differentiated thyroid cancer like PTC, with more aggressive behavior. It is about 10–30% of all well-differentiated thyroid cancer (6). Oncogenic drivers in FTC are primarily *RAS* single nucleotide alterations (*NRAS, HRAS*, and *KRAS*) and *PAX8/PPAR*γ rearrangements. *RAS* mutations and *PAX8PPAR*γ are mutually exclusive. *RAS* mutations are found in up to 40–50% of FTC; *PAX8PPAR*γ fusions are found in 30–40% of FTC cases (7, 8). *RAS* mutations and *PAX8PPAR*γ fusions are not found in cPTC but are common in FV-PTC. Molecular alterations, mutations, and/or gene copy number variations of the PI3K/Akt pathway have been identified in FTC. Genetic copy gain of *PIK3CA* has a high prevalence in FTC (24%) (9). PTEN silencing by inactivating mutations or epigenetic changes also occur in some FTC cases (10, 11).

### Poorly Differentiated Thyroid Cancer

PDTC is characterized by the absence of classic nuclear features of PTC, high mitotic activity, and tumor necrosis. Patients with PDTC often present vascular invasion lymph node metastasis, extrathyroidal extension, and sometimes distant metastases (12, 13).

Many cases of PDTC are found in patients who have a history of well-differentiated thyroid carcinoma. Sometimes, foci of WDTC coexist with PDTC, suggesting dedifferentiation of the initial tumor. PDTC has a higher mutations rate compared to WDTC. *RAS* and *BRAF* mutations are found in 20–50% and up to 35% of PDTC cases, respectively (14). Genetic alterations that characterize PDTC and are associated with tumor aggressiveness are *TERT* promoter (20–50%) mutations and *TP53* mutations (10–35%) that co-occur with *RAS* and *BRAF* mutations (15, 16). Other genetic alterations have been reported in PDTC such as *CTNNB1* and *EIF1AX* mutations and *ALK* rearrangements (15, 17). Somatic copy number alterations are common in PDTC with a gain of chromosome 1q, and loss of chromosomes 1p, 13q, 15q, and 22q (15).

### Anaplastic Thyroid Carcinoma

Anaplastic thyroid cancer (ATC) is a rare form of thyroid cancer with an extremely aggressive tumor behavior and uniformly lethal. ATC represents 1.7% of all thyroid cancer cases (18). ATC has a rapid progression and 75% of the patients present distant metastasis often in the lungs, bones, and the brain (19). ATC has a poor prognosis and accounts for 20–50% of all deaths from thyroid cancer (20) with a median survival of 3–6 months. ATC is refractory to current conventional treatments with radioiodine ablation and chemotherapy. Therefore, there is an urgent need for novel therapies for ATC. The better understanding of the molecular pathogenesis of ATC has allowed the discovery of new therapeutic strategies. Twenty to fifty percent of undifferentiated tumors are associated with WDTC suggesting and acquisition of additional genetic alterations leading to cancer dedifferentiation and acquisition of more aggressive phenotype (21). *BRAF* (20–45%), *TP53* (up to 70%), and *RAS* (20–40%) mutations are the most frequent alterations in ATC (22). In addition, *TERT* promoter mutations have been associated with *BRAF* mutation and more aggressive tumors. Many additional alterations implicated in the aggressive behavior of ATC have been reported. These alterations involve *EIF1AX, NF1, KMT*, and genes in the PI3K/Akt pathway (15). ATC cells are positive for keratin, vimentin, and N-cadherin but negative for thyroid transcription factor-1, thyroglobulin, and NIS.

### B- Treatment Modalities of Thyroid Cancer

The treatment of patients with thyroid cancer is often multidisciplinary involving surgeons, endocrinologists, medical oncologists, and nuclear medicine radiologists. Surgery remains the initial treatment of choice in patients with resectable thyroid cancer. In patients with metastatic differentiated thyroid cancer, total thyroidectomy with or without compartment-oriented lymph node dissection is still recommended to facilitate the use of radioiodine therapy and to reduce locoregional morbidity from tumor invasion. The extent of the initial thyroidectomy in patients with low-risk, differentiated thyroid cancer (tumor size smaller than 4 cm. without preoperative evidence of extrathyroidal invasion or detectable adenopathy) remains controversial as a thyroid lobectomy may provide a comparable outcome to a total thyroidectomy (1). If lymph node metastases are detected, compartment-oriented therapeutic lymph node dissection is recommended. Prophylactic central neck lymph node dissection should be performed in patients with high-risk features for nodal metastasis such as T3-T4 tumors (1).

#### Radioactive Iodine Ablation

The post-surgical management includes the RAI ablation especially for patients with high risks for tumor recurrence, patients with metastatic disease, and patients with persistent or recurrent disease. The benefit of RAI has been demonstrated in patients with a high risk for recurrence (23). RAI decreases the risk of recurrence and disease-related mortality. However, RAI for low-risk patients is controversial as there are no convincing data demonstrating its effectiveness and clinical benefit. Patients who have an excellent response after surgery, defined as undetectable thyroglobulin, do not require RAI ablation (1). High level of serum TSH by thyroid hormone withdrawal or by the administration of recombinant TSH is required to increase the uptake of radioactive iodine (I-131) in the tumor tissue by re-inducing the expression of the sodium iodine symporter (NIS) in follicular cells (24).

#### TSH Suppression

Thyroid hormone replacement after surgical resection is necessary to replace endogenous hormones and to inhibit tumor growth by maintaining low TSH level and thus reducing the risk of recurrence (25–27). The clinical benefit of TSH suppression depends on the aggressiveness of the tumor. Recent ATA guidelines recommend TSH suppression for patients with high risk (TSH goal: <0.1 mIU/L) and intermediated risk (TSH goal: 0.1–0.5 mIU/L) thyroid cancer. However, no data are demonstrating the benefit of low serum TSH level in patients with low-risk tumors after thyroidectomy (28).

#### Treatment of Advanced Thyroid Cancer

Standard therapy with surgical resection and radioactive iodine ablation fails in about 10% of differentiated thyroid cancer and all ATC. Therefore, a multimodal therapeutic approach that includes standard therapy and cytotoxic chemotherapy has been used for patients with advanced thyroid cancer but showed very limited efficacy. The significant scientific advances in the molecular characterization of thyroid cancer raised the use of more targeted therapies for advanced cancers. such as tyrosine kinase inhibitors, anti-angiogenic drugs. Recently, several novel drugs that target cell proliferation, angiogenesis, apoptosis, immunosuppression, metabolomic reprogramming, and epigenetic changes have been tested.

Somatic mutations in thyroid cancer cells lead to the activation of the MAPK and PI3K/Akt pathways. *In vitro* and preclinical studies showed an anti-tumor effect of agents targeting these pathways. BRAF^V600E^ inhibitor, vemurafenib, and MEK inhibitor selumetinib, demonstrated a reduction of the phosphorylation of ERK, increased cell cycle arrest and inhibition of tumor growth and metastasis in cell lines and *in vivo* models with MAPK activation (29–32). The growing body of evidence demonstrating the involvement of PI3K/Akt in thyroid carcinogenesis and drug resistance led to the discovery of several agents targeting the key members of this cascade. Inhibitors such everolimus and Torin-2 repressed tumors growth and cancer progression *in vivo* (33–35). Multi-kinase inhibitors targeting highly expressed tyrosine kinases (TK) in thyroid cancer cells, demonstrated a promising anti-tumor activity *in vitro* and *in vivo*. Due to its high affinity to VEGFR and FGFR, lenvatinib showed a high anti-angiogenic effects in thyroid cancer mouse model (36).

In addition to targeted therapies, immunotherapy by targeting PD-/PDL-1 axis showed a reduction of tumor growth in several pre-clinical models of aggressive thyroid cancer. Furthermore, inhibition of PD-1/PDL-1 demonstrated synergistic effects with BRAF^V600E^ inhibitor (37–39). Recent advances made in the field of cancer cell metabolism using targeted and untargeted approaches have provided data demonstrating the role of the metabolic reprogramming in carcinogenesis. The metabolic reprogramming has also been associated with genetic alterations (40). The dependence of cancer cells on certain metabolic pathways opens new avenues for the targeting of the altered metabolic pathway in patients with thyroid cancer. However, no clinical trials of drugs targeting cancer metabolic reprogramming have been initiated in patients with thyroid cancer. The following paragraph will discuss the major therapeutics strategies that have been proposed for advanced thyroid cancer.

## Targeted Therapies Against MAPK pathway

Mitogen-Activated Protein Kinase (MAPK) pathways connect extracellular signals to the network that controls cell proliferation, motility, and cell death. Thyroid cancer often presents genetic alterations that activate the MAPK pathway. These mutations include point mutations in *BRAF* and *RAS* or RET/PTC translocations (41–45). These mutations have been widely studied as a new therapeutic target in advanced thyroid cancer.

Vemurafenib and dabrafenib are BRAF^V600E^ inhibitors that function as ATP-competitive inhibitors. Vemurafenib has a much higher selectivity for mutated BRAF compared to wild type BRAF (46). A multicenter, phase I study of vemurafenib, included three patients with *BRAF*^V600E^ mutated RAI-refractory-metastatic thyroid cancer (47) (Table 1), treated with different doses of vemurafenib at 240–360 mg twice daily, and later increased to 720 mg twice a day. One patient had a partial response with a 31% reduction of the target lesion in the lungs, and the other two patients had stable disease (9 and 16% reduction of the target lesion). The progression-free interval (the time from the first day of treatment to the date of the first disease progression or date of death) for the three patients were 11.4, 11.7, and 13.2 months, and the overall survival was 15, 21, and at least 31.7 months. The adverse events were similar to those were reported in a phase I study of patients with metastatic melanoma.

**Table 1:** A table summarizing the recent clinical trials with targeted therapies that showed clinical benefits.

**Drug/targeted therapy**	**Thyroid cancer**	**N**	**ORR**	**OS, DFS**	**References**
Vemurafenib	RAI-refractory-metastatic thyroid cancer with *BRAF*^V600E^ mutation	3	1 patient had a PR, and two patients had SD	time to disease progression: 11.4, 11.7, and 13.2 mo OS: 15, 21, and at least 31.7 mo	(47)
Vemurafenib	recurrent or metastatic RAI-refractory papillary thyroid with *BRAF*^V600E^ mutation	26 22	38.5% of the patients had PR and 35% had stable disease. 27.3% of the patients had a PR and 27.3% had SD	No VEGFR-inhibitor: median PFS: 18.2 mo median OS: not reached Prior VEGFR-inhibitor: median PFS: 8.9 mo Median OS: 14.4 mo	(48)
Dabrafenib	metastatic or unresectable PTC with *BRAF*^V600E^ mutation	10	20% had a PR and 40% had SD treatment		(49)
Dabrafenib and Trametinib	BRAF^V600E^-mutated undifferentiated thyroid cancer	16	One patient (16%) had a CR and 10 patients (63%) had a PR	12-month estimates of PFS: 79%, and OS: 80%, respectively.	(50)
Pazopanib and trametinib	Advanced thyroid cancer RAI refractory	12	50% of the patients had SD and 33% had a PR	Median PFS: 10.7 mo Median OS: 29.3 mo	(51)
Selumetinib	RAI-refractory thyroid cancer	32	1 patient (3%) had a PR, 54% had SD	Median PFS: 32 weeks Median PFS for patients with *BRAF*^V600E^: 33 weeks vs 11 weeks for patients with WT *BRAF*	(52)
Selumetinib	metastatic thyroid cancer	20	Of the 8 patients treated with radioiodine, 5 had PR and and 3 had SD		(53)
everolimus	RAI-refractory thyroid cancer	33 (DTC) 7 (ATC)	1 patient (3%) had PR and 27 patients (82%) had SD one patient (14.2%) had near-CR and one patient (14.2%) had SD	Median PFS was 12.9 months Median OS was not reached	(54)
everolimus	advanced thyroid cancer	28	17 patients (65%) showed SD	Median PFS: 9 mo Median OS: 18 mo	(55)
Sorafenib	advanced or metastatic thyroid cancer	207	12.2% of the patients had PR	Median PFS: 20.5 mo for *BRAF*^V600E^-Sorafenib group vs 8.9 months *BRAF*^WT^-sorafenib group and 5.5 months for mutated *RAS*-sorafenib group vs 10.8 months for wild-type *RAS*-sorafenib group)	(56)
Lenvatinib	patients with RAI-refractory differentiated thyroid cancer	58	50% of the patients had PR 43% of the patients had SD and 28% had a durable SD.	median PFS was 12.6 mo median OS could not be estimated	(57)
Lenvatinib	RAI-refractory progressive thyroid cancer	261	4 patients (1.5%) had CR 165 patients (63.2%) had PR	Median PFS: 18.3 mo	(58)

Based on these observations, a non-randomized, multicentric phase 2 trial was initiated. Patients 18 years or older, with recurrent or metastatic RAI-refractory papillary thyroid cancer positive for *BRAF*^*V*600*E*^ mutation, with the measurable disease according to RECIST criteria, and evidence of progression in the last 14 months was included. Patients who have previously been treated with BRAF or MEK inhibitors were excluded. The enrolled patients were divided into two groups, cohort 1; with patients who had never received a multikinase inhibitor targeting VEGFR and cohort 2 with participants who had been treated previously with a VEGFR inhibitor. All patients received vemurafenib 960 mg twice a day (48) (Table 1). 38.5% of the 26 patients who had never been treated with the anti-VEGFR inhibitor, showed a partial response and 35% of the participants had stable disease. In this cohort, the median duration of response was 16.5 months, and median PFS was 18.2 months. In the cohort of 22 patients who had received VEGFR-inhibitor, 27.3% showed partial response and 27.3% had stable disease for at least 6 months. For this cohort, the median duration of response was 7.4 months, median PFS was 8.9 months and median OS was 14.4 months. Fifty-five percent of the patients died after a median follow up of 12 months. Patients who have never received anti-angiogenic multikinase inhibitor showed a longer progression-free survival compared to patients who received vemurafenib as a second line of treatment. Approximately two-thirds of patients developed grade 3 and grade 4 adverse events. Dose reduction was required in 48–58% of patients. Twenty-seven percent of patients in TKI-naïve cohort required discontinuation of vemurafenib due to adverse events. Twenty percent of patients who were treated previously with multikinase inhibitors developed cutaneous squamous cell carcinoma, 8% of patients in cohort 2 had cerebrovascular accident, and 8% of patients in cohort 1 had a squamous cell carcinoma (48) (Table 1).

These findings demonstrated an anti-tumor effect of vemurafenib in patients with progressive, *BRAF*^*V*600*E*^-positive RAI-refractory papillary thyroid cancer who had never been treated with a multikinase inhibitor and who have received vemurafenib as the first line of treatment.

Dabrafenib is a selective and a potent ATP competitive inhibitor of mutated BRAF^V600E^ (IC50 0.5 nM for V600E). A clinical trial with 10 patients with *BRAF*^V600E^ mutation, metastatic, or unresectable PTC, who have never been exposed to BRAF or MEK inhibitors, were treated with Dabrafinib, 150 mg twice daily. Fifty percent of participants had cPTC, 40% had a TC-PTC, and 10% had a clear cell papillary thyroid cancer. Sixty percent of the patients showed new radioiodine uptake. Six months after radioiodine treatment following dabrafenib, two patients showed a partial response (40 and 60% reduction of the target lesion) and the remaining four patients had stable disease at 3 months follow-up. The most common adverse effects observed were skin lesions, fatigue, electrolyte abnormalities, and palmar-plantar erythrodysesthesia. However, there was no dose reduction during the study (49) (Table 1). Despite the re-differentiation observed in 60% of the patients treated with Dabrafenib, these findings suggest that a combination therapy targeting BRAF^V600E^ and MEK would have a greater clinical benefit.

Given the numerous studies supporting the effectiveness of the combination therapy of dabrafenib with the MEK inhibitor trametinib in melanoma, a similar combination therapy was given to patients with undifferentiated thyroid cancer in a multicenter an open-label non-randomized phase II trial (50) (Table 1). Patients with the age of 18 or older, with measurable disease, and with *BRAF*^V600E^ mutated tumors were enrolled in the trials. Patients who have been previously treated with BRAF and/or MEK inhibitors were excluded from the study. Combination therapy of dabrafenib and trametinib showed a significant anti-tumor activity and 69% overall response rate with 1 patient with a complete response. Twelve months duration of response was 90%, PFS was 79%, and OS was 80%. The most common adverse effects of the treatment were fatigue, pyrexia, and nausea. Adverse effects that required discontinuation were reported in 44% of the patients with ATC. This study demonstrated a great clinical benefit in metastatic undifferentiated thyroid cancer. Dabrafenib and trametinib were approved by the FDA for ATC because of the efficacy in ATC patients with *BRAF*^V600E^ mutation.

In a recent open label-multicenter clinical study, patients with advanced thyroid cancer were treated with a combination of trametinib with VEGFR tyrosine kinase inhibitor; pazopanib. Increased level of VEGF and VEGFR1 has been associated with a higher risk of recurrence and the presence of locoregional and distant metastases (44, 59, 60). Patients enrolled in the study must have a progressive disease, RAI-non avid lesions and a measurable disease to evaluate the response rate. Out of the 13 patients with DTC enrolled in the study, 33% showed a partial response and 50% had stable disease. The median follow-up was 37 months. Median progression-free survival and overall survival were 10.7 and 29.3 months. Although trametinib and pazopanib combination therapy showed a high response rate, the lack of a single agent arms does not allow the comparison of the combination therapy vs. single agent (51) (Table 1).

Selumetinib is a potent non-ATP competitive MEK1/2 inhibitor. Selumetinib showed a preferential anti-tumor activity in *BRAF*^V600E^ mutated thyroid cancer cell lines (31, 32). In an open-label phase II clinical trial including patients with RAI-refractory thyroid cancer with a known tumor genotype and a progressive disease in the last 12 months who have never been exposed to any TKI targeting the MAPK pathway were enrolled. Selumetinib was given at a dose of 100 mg twice daily. Of 32 evaluable patients, only one patient had a partial response and 21 patients had stable disease. Patients with *BRAF*^V600E^ tumors had a longer median PFS compared to patients with WT *BRAF* tumors. These findings were consistent with the preclinical data demonstrating a higher anti-tumor activity of selumetinib in *BRAF*^V600E^ mutated cells. The most common adverse effects that were reported are fatigue, diarrhea, and rash. Of 39 enrolled patients, 12 patients had dose reduction and 6 patients discontinued the treatment due to severe adverse effects (52) (Table 1).

Another clinical study of selumetinib was performed in a cohort of 24 patients with metastatic differentiated thyroid cancer treated at a dose of 75 mg twice a day. The endpoints were the refractoriness to RAI and cancer cell redifferentiation. Selumetinib treatment increased the iodine-124 uptake on PET-CT in all patients with *N-RAS* mutations (5) and in four out of the nine patients with *BRAF* mutation. Of all eight patients who qualified for RAI therapy, five patients had a partial response (one patient with *BRAF*^V600E^ mutation) and three had stable disease and all showed a reduction of thyroglobulin levels. These results suggest selumetinib is more effective in RAI-refractory thyroid cancer with *NRAS* mutation compared *BRAF*^V600E^ mutated tumors. Selumetinib by inhibiting MAPK pathway re-differentiate thyroid cancer cells and increases iodine uptake (53) (Table 1).

A single-arm multicenter two-stage phase II clinical trial in The United kingdom, aiming at evaluating the efficacy of selumetinib followed by RAI therapy in patients with RAI-refractory thyroid cancer is currently conducted and recruiting patients (61).

### Targeting of PI3K/Akt Pathway

The PI3K/Akt pathway plays a crucial role in thyroid carcinogenesis, cell dedifferentiation, invasion, and metastasis. The three main players of these pathways are PI3K, Akt, and mTOR. Activating mutations and copy gain in the gene coding for the catalytic subunit of PI3K (PIK3CA), and inactivating mutations and hypermethylation of the tumor suppressor gene PTEN have been identified in advanced DTC especially follicular thyroid cancer and ATC. These genetic and epigenetic alterations result in angiogenesis, drug resistance, and thyroid cancer progression and metastasis. The targeting of the PI3K/Akt/mTOR has been an active area of cancer research. Several inhibitors showed promising anti-tumor activity in thyroid cancer cells *in vitro* and *in vivo*.

A recent open-label phase II clinical trial evaluated the efficacy of everolimus in RAI-refractory thyroid cancer. Patients of the age of 18 or older with progressive, advanced, or metastatic thyroid cancer who had received up to TKI were enrolled in the study and treated with 10 mg of everolimus daily. Out of the 40 patients with follicular cell-origin thyroid cancer, 35% had PTC and 17% had ATC. 72.5% of patients with follicular cell-origin cancer had a stable disease and only 5% had a partial response. One of the patients who had a partial response had ATC with PI3K/Akt/mTOR mutations. The results do not allow a correlation between the response to everolimus and the genomic signature of the tumor, mainly because of the small sample size of each genomic group (54) (Table 1).

In another phase II clinical trial assessing the efficacy of everolimus in a cohort of patients with advanced thyroid cancer, everolimus was administered at the dose of 10 mg daily. Sixty-five percent of patients with differentiated thyroid cancer showed a stable disease as the best response. Forty-six percent of the patients required a dose reduction because of the toxicity of everolimus. Analysis of the somatic mutations in patients' samples did not show any association between mutation status and response to everolimus (55) (Table 1).

These two studies demonstrated the modest anti-tumor activity of everolimus and the stabilization of the disease. However, both trials failed to demonstrate and an association between the mutation status of the tumor and the response to the drug in patients with advanced thyroid cancer.

### Anti-angiogenic TKI

#### Sorafenib

Sorafenib is a multikinase inhibitor that targets VEGFR 1,2 and 3, RET, FLT3, c-Kit, BRAF, and BRAF^V600E^. It is approved for patients with advanced, iodine-refractory thyroid cancer. Several studies have evaluated the efficacy of sorafenib in advanced thyroid cancer. A phase III placebo-controlled multicenter study assessed the progression-free survival in patients with advanced or metastatic thyroid cancer treated with sorafenib. Patients with age of 18 or older, with advanced or metastatic RAI-refractory thyroid cancer who had progressed in the last 14 months were enrolled. Patients who had received any targeted therapy in the past or chemotherapy were excluded. Sorafenib was given at a daily dose of 800 mg. Four hundred and nineteen patients were enrolled in the trial, 207 patients in the sorafenib group and 210 in the placebo group. The median PFS in sorafenib group was significantly longer than that of placebo group (10.8 months for sorafenib group compared to 5.8 months for the placebo group, *p* < 0.001). PFS improvement was observed in all groups irrespective of clinical or demographic features or mutation status. Subgroup analysis showed a significantly longer PFS in patients who had *BRAF*^V600E^ and *RAS* mutated thyroid cancers compared to those with tumors harboring respective mutated genes receiving placebo (20.5 months for *BRAF*^V600E^ -Sorafenib group vs. 9.4 months *BRAF*^V600E^ -Placebo group and 5.5 months for mutated *RAS*-sorafenib group vs. 3·5 months mutated *RAS-*Placebo group). The significantly longer PFS was observed in patients with *BRAF*^WT^ and *RAS*^WT^ thyroid cancer treated with sorafenib compared to that of patients with tumors harboring respective wild-type genes receiving placebo (8.9 months *BRAF*^WT^-sorafenib group vs. 3.8 months for *BRAF*^WT^-Placebo group and 10.8 months for wild-type *RAS*-sorafenib group vs. 5.8 months in the wild-type *RAS*-placebo group). Although patients with *BRAF* mutation showed the longest PFS, neither mutation was predictive of sorafenib response. However, there was no significant difference in the OS between sorafenib and placebo-treated group likely because 71.2% of patients in the placebo group crossed over to receive sorafenib. 12.2% of the sorafenib treated patient showed a partial response vs. 0.5% in the placebo group. Thirty-seven percent of the patients enrolled in the sorafenib group experienced serious adverse events. The most common grade 3 and 4 adverse effects in sorafenib treated group were hand-foot skin reaction, diarrhea, rash or desquamation, and hypertension. One death in the sorafenib group was drug-related (56) (Table 1). These findings demonstrated a significant improvement of PFS in patients with RAI-refractory thyroid cancer treated with sorafenib. FDA approved sorafenib for the treatment of locally recurrent or metastatic, progressive differentiated, iodine-refractory, thyroid cancer in 2013.

#### Lenvatinib

Lenvatinib is a multikinase inhibitor, targeting VEGFR1-3, FGFR, PDGFR, RET, and KIT (45, 62, 63). An open-label single-arm phase 2 clinical trial was conducted to evaluate the anti-tumor activity of lenvatinib in advanced thyroid cancer. Fifty-eight patients with RAI-refractory differentiated thyroid cancer who have not received any antiangiogenic targeted therapy in the last 30 days prior to lenvatinib administration were enrolled. Fifty percent of the patients had a partial response, with no significant difference between patients with prior VEGFR- targeted therapy vs. not. Forty-three percent of the patients had stable disease (≥7 weeks) and 28% had a durable stable disease (≥23 weeks). Most common adverse effects observed were hypertension, weight loss, diarrhea, proteinuria, fatigue nausea, and loss of appetite. Dose interruptions, reduction, and study drug withdrawal occurred in 74, 66, and 26% of the enrolled patients, respectively. Two patients died from drug-related toxicity (57) (Table 1).

A phase 3 clinical trial with a primary end-point the progression free survival in patients with RAI-refractory progressive thyroid cancer treated with lenvatinib or placebo, demonstrated a significantly longer PFS in patients who received lenvatinib. The median progression survival in the lenvatinib group was 18.3 vs. 3.6 months in the placebo arm. As expected, patients in the lenvatinib group experienced more adverse effects. The most common AEs observed were hypertension, diarrhea, fatigue, and decreased appetite and 6 out of the 20 deaths in the lenvatinib group were drug-related (58) (Table 1). FDA approved lenvatinib for the treatment of advanced, progressive differentiated, iodine-refractory, thyroid cancer in 2013.

### Immunotherapy

The interactions between the tumor microenvironment and cancer cells are involved in cancer progression. The increasing findings regarding the mechanisms underlying the ability of cancer cells to escape the immune response surveillance have made the immune system a new promising therapeutic target in oncology.

Analysis of the Immunoscore, a method to estimate the prognosis of patients with cancer based on the infiltrative immune cells in cancer, of 505 patients with thyroid cancer, revealed a significant negative correlation between thyroid differentiation score and immunosuppressive markers such as CTLA-4 and PD-L1. These immunosuppressive markers were significantly higher in *BRAF*^V600E^-mutated tumors compared to wild-type tumors (64). Increased PD-L1 expression has been demonstrated in several solid tumors and has been associated with poor prognosis (65, 66). Tumors cells harboring PD-L1 expression inhibits T cell activation in the tumor microenvironment thus protecting tumors cells from the immune response. Analysis of the prognostic potential of PD-L1 in 185 PTC and 66 benign nodules, showed that increased expression of PD-L1 in thyroid cancer is associated with a higher risk of recurrence and shorter DFS (67). These findings suggest that the targeting of PD-L1 could be an effective therapy in advanced thyroid cancer.

In a phase Ib clinical trial assessing the safety and the antitumor activity of the anti-PD-1 antibody pembrolizumab, 22 patients with locally advanced or metastatic differentiated thyroid cancer, positive for PD-L1 expression, who have never received anti-cancer monoclonal antibody therapy in the last 4 weeks before treatment, or targeted therapy in the last 2 weeks prior the treatment. Patients participating in the trial received 10 mg/kg of pembrolizumab every 2 weeks for 24 months. Two patients with PTC had a PR after 4 and 5 months of treatment, and response duration of 20 and 8 months. Fifty-seven percent of the patients with FTC and 60% of patients with PTC had a stable disease with a median duration of 7 months. PFS rate at 6 and 12 months were 59 and 36%, respectively (68). These observations showing a clinical benefit of pembrolizumab suggest that targeting PD-1 is a good therapeutic strategy for advanced thyroid cancer.

In addition, a combination of PLX4720, a *BRAF*^V600E^ inhibitor and anti-PD-L1/PD-1 antibody in a preclinical model of ATC, reduced the tumor growth, and increased in tumor CD8^+^ cytotoxic T cells, FoxP3^+^ Tregs, and NK cells, thus demonstrating an induction of the immunosuppressive tumor microenvironment. Taken together, these findings suggest that a combination therapy of BRAF^V600E^ inhibitor and anti- PD-1/PD-L1 antibody is a promising therapeutic strategy for metastatic thyroid cancer.

## Conclusions

The treatment of advanced and metastatic iodine-refractory thyroid cancer remains very challenging. Recent discoveries in the molecular pathogenesis of thyroid cancer have led to major advances in the treatment and management of aggressive tumors. Preclinical and clinical studies with targeted therapies and immune therapies have demonstrated a significant clinical benefit. Further studies are needed to improve these therapeutic strategies to offer a better treatment for patients with advanced and metastatic thyroid cancer.

## Author Contributions

All authors have contributed to the preparation of this manuscript. All authors have read and approved the manuscript.

## Conflict of Interest

The authors declare that the research was conducted in the absence of any commercial or financial relationships that could be construed as a potential conflict of interest.
